# Moss inhabiting flea beetles of the West Indies III: *Erinaceialtica*, a new genus from Hispaniola (Coleoptera, Chrysomelidae, Galerucinae, Alticini)

**DOI:** 10.3897/zookeys.955.53644

**Published:** 2020-08-05

**Authors:** Alexander S. Konstantinov, Adelita M. Linzmeier

**Affiliations:** 1 Systematic Entomology Laboratory, USDA, c/o Smithsonian Institution, P. O. Box 37012, National Museum of Natural History, Washington, DC 20013-7012, USA National Museum of Natural History Washington United States of America; 2 Universidade Federal da Fronteira Sul, Rua Edmundo Gaievski, 1000, P. O. Box 253, 85.770-000, Realeza, PR, Brazil Universidade Federal da Fronteira Sul Realeza Brazil

**Keywords:** bryophyte, Monoplatina, moss cushions, new genus, new species, West Indies

## Abstract

*Erinaceialtica* Konstantinov & Linzmeier, a new genus of moss inhabiting flea beetles, containing seven species from the Dominican Republic and Haiti is described and illustrated. Five species are new (*E. gabbysalazarae*, *E. janestanleyae*, *E. rickstanleyi* (the type species), *E. rileyi*, and *E. thomasi*) and two species are transferred from *Aedmon*: *A. albicincta* (Blake) and *A. hugonis* (Blake) **comb. nov.** The new genus is compared to *Aedmon* Clark, *Apleuraltica* Bechyne, *Andersonaltica* Linzmeier & Konstantinov, *Distigmoptera* Blake, and *Ulrica* Scherer. Keys to *Erinaceialtica* and related genera and to *Erinaceialtica* species are provided.

## Introduction

Moss cushions in the West Indies, and in Hispaniola in particular, continue to reveal previously unknown flea beetles and allow proper classification of already described ones. Specimens that form a foundation of this paper were collected on the island of Hispaniola. The ones that we have collected in 2005, 2006, and 2014 were extracted from moss cushions the same way as the other moss-inhabiting flea beetles were (for methods see Linzmeier and Konstantinov 2020). However, it remains unknown if *Erinaceialtica albicincta* (Blake, 1945) and *E. hugonis* (Blake, 1943a) collected by Darlington in Haiti and the Dominican Republic, respectively, came from moss cushions, although they both were found at significant altitude where moss is generally available. In addition, it seems that *E. rileyi* and *E. thomasi* did not come from moss cushions as well. There is a chance that they were sifted out of leaf litter as their collectors (C. O’Brien and M. Thomas) sifted leaf litter regularly. Species of a number of flea beetle genera which occur in moss are also known to live in leaf litter [e.g. *Andersonaltica* Linzmeier & Konstantinov (Konstantinov et al. in press), *Benedictus* Scherer (Sprecher-Uebersax et al. 2009, *Ulrica* Scherer (Konstantinov and Konstantinova 2011)]. Even if beetles were picked up in sweep net, that would not be the first example of catching moss living flea beetles with a net [e.g. *Menudos chamorrae* (Konstantinov and Konstantinova 2011)].

The three species, for which association with moss is established, are the very rare examples of flea beetles that live in moss cushions but have fully developed wings and elytra that are free with fully developed humeral calli. Another example is *Distigmoptera borealis* Blake, 1943b (Konstantinov et al. 2019). However, ability of *Erinaceialtica* species to fly have not been verified. As has been noticed previously, having fully developed wings, but shortened metasternites (e.g. *Nicaltica*Konstantinov et al. 2009) may be an indication that the flight muscles are reduced. *Erinaceialtica rickstanleyi* has such a metasternite (Fig. 32). Although these beetles have wings (Fig. 2), they probably cannot fly, as in the case of some Neotropical cicindelines (Zerm and Adis 2002).

## Material and methods

Dissecting techniques and terminology for most internal and external structures follow Konstantinov (1998). In addition, terminology for adult thoracic structures and ridges follows Lawrence and Slipinski (2013), Lingafelter and Konstantinov (2000), and McHugh et al. (1997). Specimen labels are sited verbatim, according to the format justified previously (Konstantinov 1998, Konstantinov and Lingafelter 2002, and Konstantinov et al. 2011). Specimen observations were made with a Zeiss Stemi SV11 Apo microscope. Digital photographs of morphological structures were taken with Axio Zoom V16 microscope and AxioCam HRC digital camera attached to it and with AxioCam HRC Zeiss attached to Leitz Diaplan compound microscope. Additional images were taken with Macropod Pro photomacrography system (Macroscopic Solutions, LLC, Tolland, CT).

The beetles are deposited in the following collections: National Museum of Natural History, Smithsonian Institution, Washington, DC (USNM); E. Riley collection (ERPC); and Museo Nacional de Historia Natural, Santo Domingo, Dominican Republic (MHND).

## Taxonomy

### Erinaceialtica gen. nov.

Taxon classificationAnimaliaColeopteraChrysomelidae

0903EFEB-6FFB-597A-8953-7307EA2A3BD8

http://zoobank.org/D8DC4FAF-5A12-454D-B8B5-11E29E5FE557

Figs 1 , 2–4 , 5–7 , 8–11 , 12, 13 , 14 , 15–19 , 20, 21 , 22 , 23–27 , 28–33 , 34–40 , 41–43 , 44, 45 , 46, 47 , 48–51 , 52–54 , Map 1

#### Description.

Body length 2.1–2.81 mm, width (widest point of elytra) 1.19–1.35 mm, height 0.97–1.13. Elytron metallic green, blue, black, or coppery, light metallic green; some parts of elytra yellow to brown. Color of pronotum almost same as elytron: metallic blue, green, or dark brown, in some species pronotum greenish when elytra blueish.

Head. Orbit as wide as transverse diameter of antennal socket with punctures dense, large, their diameter greater than distance between them. Inner margins of eye straight, nearly parallel with each other. Distance between eyes (above antennal sockets) in frontal view much greater than transverse diameter of eye (2.4 times). Supraorbital pore only slightly larger than other punctures, but different color (mostly yellowish). Vertex densely and evenly covered with round, setose punctures placed close together. Antennal sockets situated below middle of eye. Frontal ridge (from dorsal side to frontoclypeal suture) 1.58–1.87 times longer than longitudinal diameter of antennal socket; straight in lateral view; extends to level of antennal calli, does not enter in between them. Sides of frontal ridge between antennal sockets and below straight, parallel to each other. Dorsal side of frontal ridge truncate. Frontal ridge and vertex separated by antennal calli. Frontal ridge dorsally as wide as ventrally. Anterofrontal ridge tall, slopes abruptly towards clypeus. Dorsal side of anterofrontal ridge laterally of frontal ridge even, without visible convexity. Frontal ridge and anterofrontal ridge in frontal view form nearly straight angle with each other. Length (thickness) of anterofrontal ridge less than that of frontal ridge. Sides of head below eyes converging ventrally. Shape of clypeus band like. Anterior margin of labrum emarginate. Labrum with 3 pairs of setae, distributed evenly on both sides.

Head sulci and antennal calli. Midcranial suture absent. Supraorbital, orbital, supracallinal, frontolateral and suprafrontal sulci absent. Midfrontal sulcus well developed, long. Antennal calli and top of frontal ridge meet, not separated from each other. Antennal callus not entering interantennal space. Surface of antennal callus on same level as surface of vertex and frontal ridge. Length of antennal callus about as great or shorter than its width. Antennal grooves between eye and frontal ridge present.

Antenna with 11 antennomeres, apical antennomeres much wider than basal. Color of antennomeres (not counting basal antennomeres being a bit lighter than rest) different. Antennomere 5 white. Antenna not reaching half of elytron. Antennomere 1 shorter than next two antennomeres combined. Antennomere 2 globular, shorter than 3, longer than half of it, about as wide as 1, wider than 3. Antennomere 3 longer than 4. Antennomere 5 as long as 4, longer than or as long as 6. Antennomere 7 much wider than antennomeres 4 and 5 separately. Distal antennomeres robust.

Prothorax. Pronotal surface hairy. Anterolateral callosity ovoid or otherwise rounded. Anterolateral callosity: expansion beyond lateral margin slight, facing anterolaterally. Anterior setiferous pore situated about middle callosity. Anterolateral corners of pronotum projected at about same level as middle of pronotum. Sides of pronotum nearly straight with a slight lobe anterior to middle. Pronotal margins even. Base of pronotum straight, without lateral longitudinal impressions. Antebasal transverse impression on pronotum wide, shallow, poorly differentiated from rest of pronotal surface or absent. Longitudinal impressions anteriorly present in middle. Two bumps with groove between present. Lateral margin of pronotum complete. Numerous setae on lateral margin of pronotum present. Posterolateral callosity situated on corner of posterior and lateral margins. Procoxal cavities closed. Intercoxal prosternal process in lateral view more or less straight or slightly convex, in ventral view generally wide, surface concave, posteriorly much wider than in middle. Sides of intercoxal prosternal process concave, posterior end straight, extends beyond procoxae posteriorly.

Mesothorax. Scutellum present. Mesothoracic prefragma very short. Mesoventral process about as wide as long. Mesocoxal cavities transverse. Mesosternum without elevated projection in middle.

Elytra with sides nearly straight, sometimes parallel to each other. Humeral calli well developed. Elytron with basal callus. Transverse impression of elytron posteriad to humeral or basal callus deep. Oblique impression on elytron between humeral and basal calli present. Elytron with punctures arranged in striae, not in grooves. Dorsal surface covered with sparse erect and dense hairs arranged in different directions. Elytra at base wider than base of pronotum. Ridges on elytra absent. Epipleura about as wide as front femur, abruptly narrowing before apex, oblique, directed outwardly, reaches end of side of elytron, but not apex.

Metathorax. Metasternum short, about twice as long as mesosternum, anteriorly projecting forward, but not covering mesosternum, without elevated projection in middle. Posterior end of metasternum slightly swollen. Metathoracic discrimen extends a bit more than half of metasternum length. Metatergite about twice as wide as long, with full set of ridges in middle. Metendosternite with relatively short stem, slightly narrower than arms near base. Arm tendon slightly closer to middle than to arm end. Arm ends lightly sclerotized, simple.

Abdomen. Abdominal ventrites 1 and 2 not fused. Abdominal ventrite 1 slightly longer than ventrites 2 and 3 together. Abdominal ventrite 5 slightly shorter than ventrites 4 and 3 together. First abdominal ventrite between coxae without longitudinal ridges. Apex of first abdominal sternite in female evenly and narrowly rounded. Last visible tergite of female without longitudinal groove in middle.

Legs. Pro- and mesotibiae and femora not sexually dimorphic. Profemur generally cylindrical. Pro- and mesotibial spurs absent. Pro- and mesotibiae without longitudinal ridges. Apical part of middle tibia without obtuse tooth beyond middle, followed by excavation. Metafemur enlarged. Posterior edge of metafemur in males as in females. Metafemoral spring present. Metatibia in dorsal and lateral views straight. Metatibia in cross section around middle more or less cylindrical, dorsally convex. Metatibial apex flattened. Sharp edge present on dorsal side of metatibia laterally, absent medially. Metatibia with transverse ridge above insertion of tarsus. Metatibial spur situated laterally, single, simple, narrow, ending in one tooth, shorter than greatest width of metatibial apex. Metatibia ventrally at apex makes lobe on side of spine. Metatarsomere 1 attached anteriorly of apex. First protarsomere of males about as wide and long as in females. Protarsomere 3 wide, with round sides. Metatarsomere 3 longer than wide, elongate, incision absent. Metatarsomere 4 globose. Claw bifid or appendiculate.

Genitalia. Spermatheca with receptacle and pump without distinct border in between. Receptacle curved, three dimensional, longer than wide or as wide as long. Receptacle about as wide as pump. Pump with flattened end. Duct of spermatheca with coils longer than receptacle or as long as receptacle. Vaginal palpi many times longer than wide. Posterior sclerotization of vaginal palpi elongate, rounded at apex. Tignum narrow anteriorly, widens posteriorly. Median lobe of male genitalia elongate. Median lobe of male genitalia in cross section (about middle) oval or flattened.

**Map 1.**
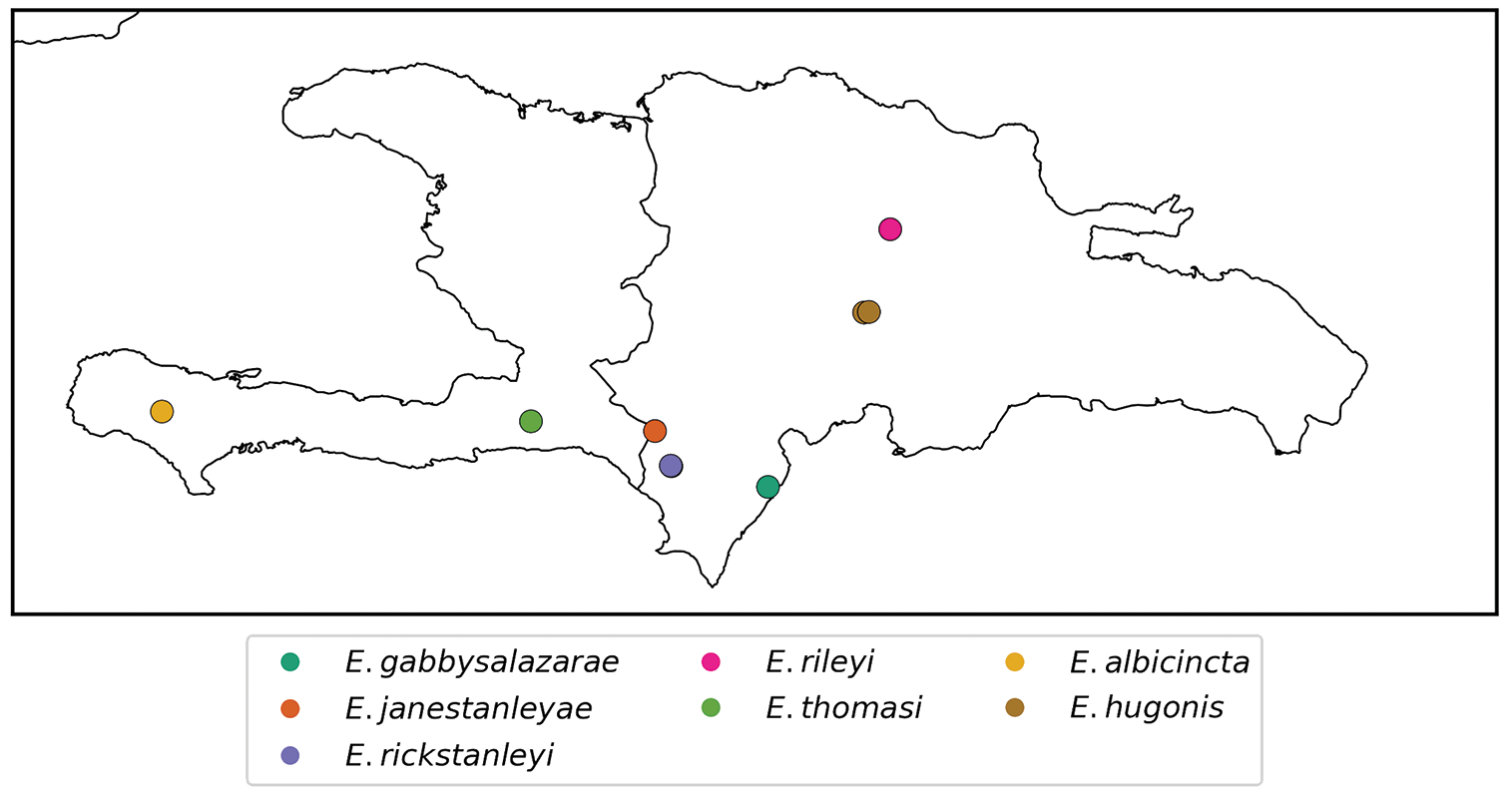
Distribution of *Erinaceialtica* species on the island of Hispaniola.

#### Type species.

*Erinaceialtica rickstanleyi* sp. nov. by present designation.

#### Etymology.

The name of this genus is a combination of two words. First is a Latin word for hedgehog – Erinacei (genitive, singular, masculine, II declension) as a reference to beetles having highly unusual setae that remind needles of a hedgehog. The second word is Altica, the name of the type genus of the Alticini.

#### Comparative diagnosis.

The first known species of *Erinaceialtica* have been described in the genus *Hadropoda* (Blake 1943a, 1945). However, while describing *E. hugonis*, Blake (1943a) noticed that this species is substantially different from West Indian congeners “and may eventually be placed in a different genus …” (page 440). She made similar observations two years later describing *E. albicincta* (Blake, 1945) suggesting that “… together they form a group that stands a little apart in the genus [*Hadropoda*] and resembles in many ways the species of the North American genus *Distigmoptera*. *H. albicincta* closely resembles *hugonis* but differs in having raised sutural margins and three warts as well as an apical tumidity on each elytron. Its elytral punctation is a little finer and the whole beetle slightly more slender” (page 89).

Classification of monoplatine genera presents significant difficulties, moss and leaf litter inhabiting and associated morphological transformations contribute to it. Nevertheless, we were able to circumscribe some distinct species groups among West Indian species that include already described genera (*Aedmon* Clark, *Andersonaltica* Linzmeier & Konstantinov, *Apleuraltica* Bechyne, *Distigmoptera* Blake, *Menudos* Linzmeier & Konstantinov, and *Ulrica* Scherer) and the one described in this paper. To facilitate their identification, we provide a key below.

Some features of *Erinaceialtica* that cannot be included in the key, but still worth mentioning are: 1) presence of subtle sexual dimorphism in the general color of the body (small difference in size of dark spots is noticed in *E. gabbysalazarae*) and width of the head (sexual dimorphism among *Menudos* species exceeds one that generally occurs in flea beetles); 2) median lobe of the aedeagus differs dramatically between species (in most genera, particularly those occurring in leaf litter or moss, median lobes are very similar and differ only in very subtle features e.g. *Andersonaltica*); 3) species are very colorful (more so than flea beetles in general), dramatically different in color of the body and appendages as well as in color and direction of the setae on pronotum and elytra; this makes species identification relatively easy based on external characters and makes it possible to identify *E. albicincta* and *E. hugonis* based on images of the types (see MCZ citations in the reference section).

### Key for Monoplatina genera of the West Indies related to *Erinaceialtica*

**Table:** 

1	Head and pronotum almost smooth with small and shallow punctures; pronotum and elytra lack hairs or with sparse hairs; pronotum without ridges	***Ulrica* Scherer** (Fig. 61)
–	Head and pronotum densely and deeply punctuated; pronotum and elytra covered with hairs; pronotum with ridges	**2**
2	Body oblong-oval, elongate, subparallel	**3**
–	Body elliptical to rounded	**5**
3	Pro- and mesotibiae bicolored with wide yellowish band in middle (band may be very vivid (Fig. 1), or subtle (Fig. 14); antennomere 5 always white; pronotum and elytra with different metallic tint	***Erinaceialtica* gen.nov.**
–	Pro- and mesotibiae without wide yellowish band in middle; antennomere 5 similar in color to other antennomeres; pronotum and elytra not metallic	**4**
4	Elytra with impression near the suture before the middle	***Distigmoptera* Blake** (Fig. 59)
–	Elytra without impression or with a very faint one near the suture before the middle	***Aedmon* Clark** (Fig. 56)
5	Basal callus poorly developed, antennae filiform with all antennomeres longer than wide; pygidium exposed	***Menudos* Linzmeier & Konstantinov** (Fig. 60)
–	Basal calli well developed; antennae with some distal antennomeres wider than long; pygidium not exposed	**6**
6	Antennal calli widely separated anteriorly; antennae clavate, antennomeres 7 to 11 forming tight club; elytra uneven, highly convex in lateral view; metatibia without transverse ridge above insertion of tarsus	***Andersonaltica* Linzmeier & Konstantinov** (Fig. 57)
–	Antennal calli longitudinal, not widely separated anteriorly; antennomeres 7 to 11 more robust forming almost a clave, with antennomeres 8 to 10 wider than long; elytra even, slightly convex in lateral view; metatibia with transverse ridge above insertion of tarsus forming a preapical dorsal projection, in lateral view	***Apleuraltica* Bechyné** (Fig. 58)


### Erinaceialtica gabbysalazarae sp. nov.

Taxon classificationAnimaliaColeopteraChrysomelidae

6AD9E45D-8586-5C2C-9646-6C545A44D3A4

http://zoobank.org/A55E8C8F-B188-42FF-8A1D-DF10875B09C1

Figs 1 , 2–4 , 5–7 , 8–11 , 12–13 , Map 1

#### Description.

Body length 2.27–2.64 mm, width 1.29–1.51 mm. Vertex dark brown, part of head below vertex mostly antennal calli and frons yellowish. Antennomere 11 slightly lighter than 9. Base of pronotum yellowish, apex dark. Sides of pronotum dark. Antebasal pronotal impression shallow, poorly defined. Elytral disc broadly yellow, except triangular spot on side and below scutellum, sides and slope of elytron, two spots at beginning of elytral slope in male and larger spot in female. Elytral apex yellowish, slightly darker than elytral disc. Ventral side of body orange. Base of pro- and mesofemora and middle of pro- and mesotibiae white. Apex of pro- and mesofemora and base and apex of pro- and mesotibiae dark brown. Basal half of metafemora dark, apical lighter. Setae on orbit bright yellow, directed ventrally. Setae on middle of vertex directed towards middle, forming a small “mohawk”. Pronotal setae directed posteriorly starting from about middle. Median lobe of aedeagus flattened, nearly straight in lateral view, complex ventrally with deep longitudinal impression from base to apex between two sharp ridges and a lobe in middle of impression; narrow before apex, widening towards it, apex itself ogival in shape.

**Figure 1.**
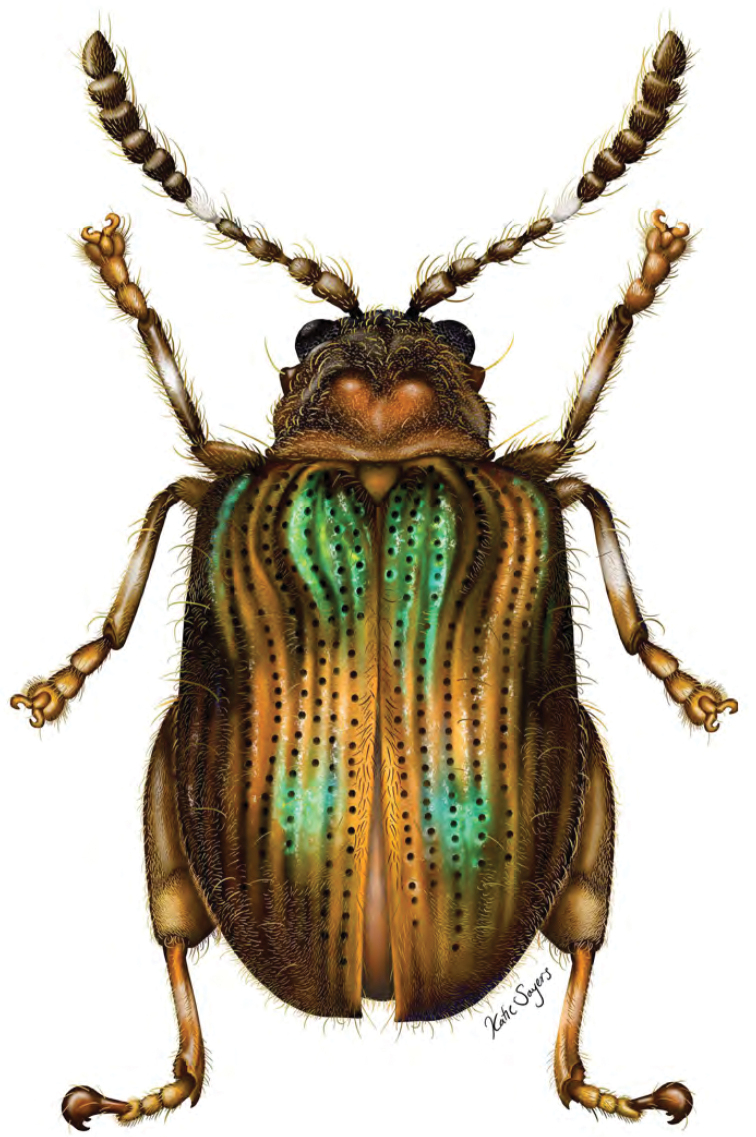
*Erinaceialtica gabbysalazarae* sp. nov., dorsal habitus (illustration by Katie Sayers, USNM and SEL scientific illustrator internship program, summer 2019).

#### Diagnosis.

*Erinaceialtica gabbysalazarae* has a uniquely shaped median lobe of the aedeagus and unique color and can be easily identified using the key at the end of the paper.

**Figures 2–4.**
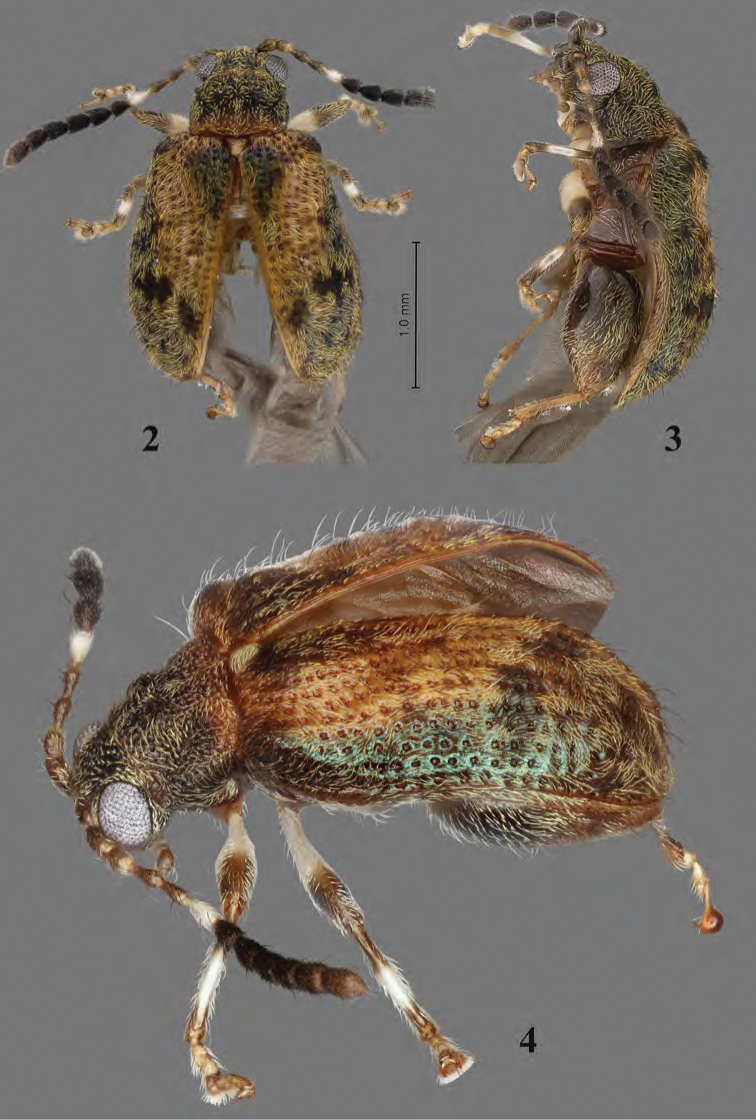
Adult *Erinaceialtica gabbysalazarae*, male **2** habitus, dorsal view **3** habitus, lateral view **4** habitus, three quarter view.

#### Habitat.

*Erinaceialtica gabbysalazarae* was collected at El Cachote forest on a rainy day in moss that was hanging from branches and growing trunks of the trees (Figs 12, 13). Altogether about 5 gallons (= 19 L) of moss was collected in a single pillowcase. Some of it was processed directly with Berlese extraction and some was sifted and then processed with Berlese.

**Figures 5–7.**
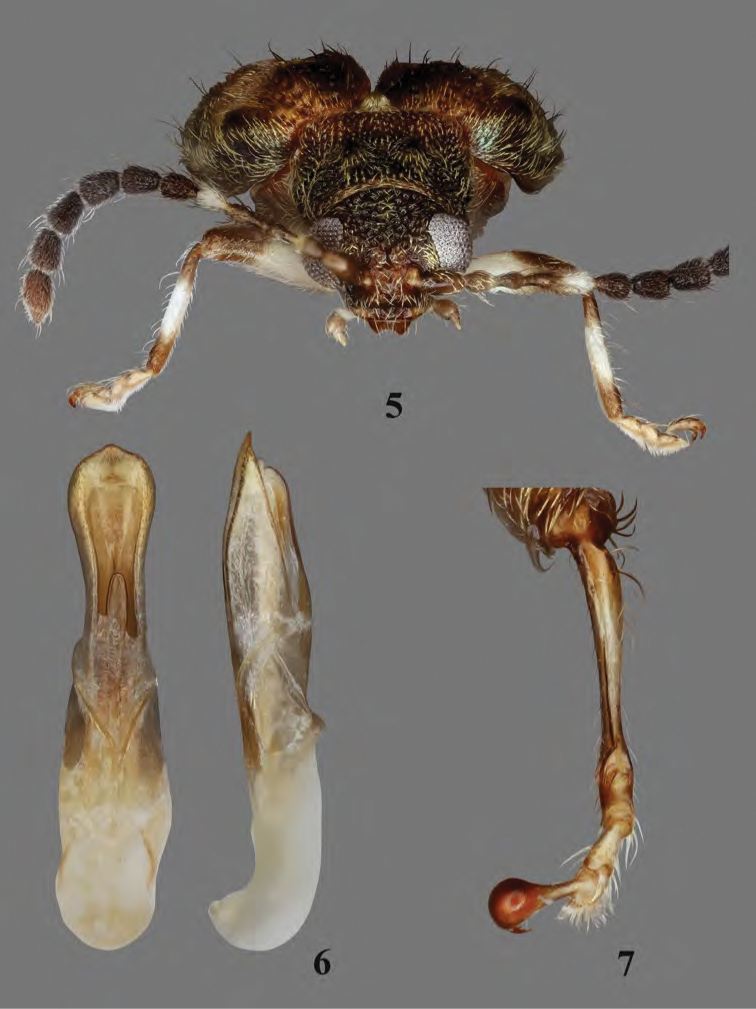
Adult *Erinaceialtica gabbysalazarae*, male **5** habitus, frontal view **6** median lobe of aedeagus, ventral and lateral views **7** metatibia, dorsal view.

#### Etymology.

The species epithet, gabbysalazarae, is a matronym in honor of Gabby Salazar of Bethesda, Maryland in appreciation for comradery and companionship during collecting trips to the Dominican Republic, which she documented in numerous photos.

**Figures 8–11.**
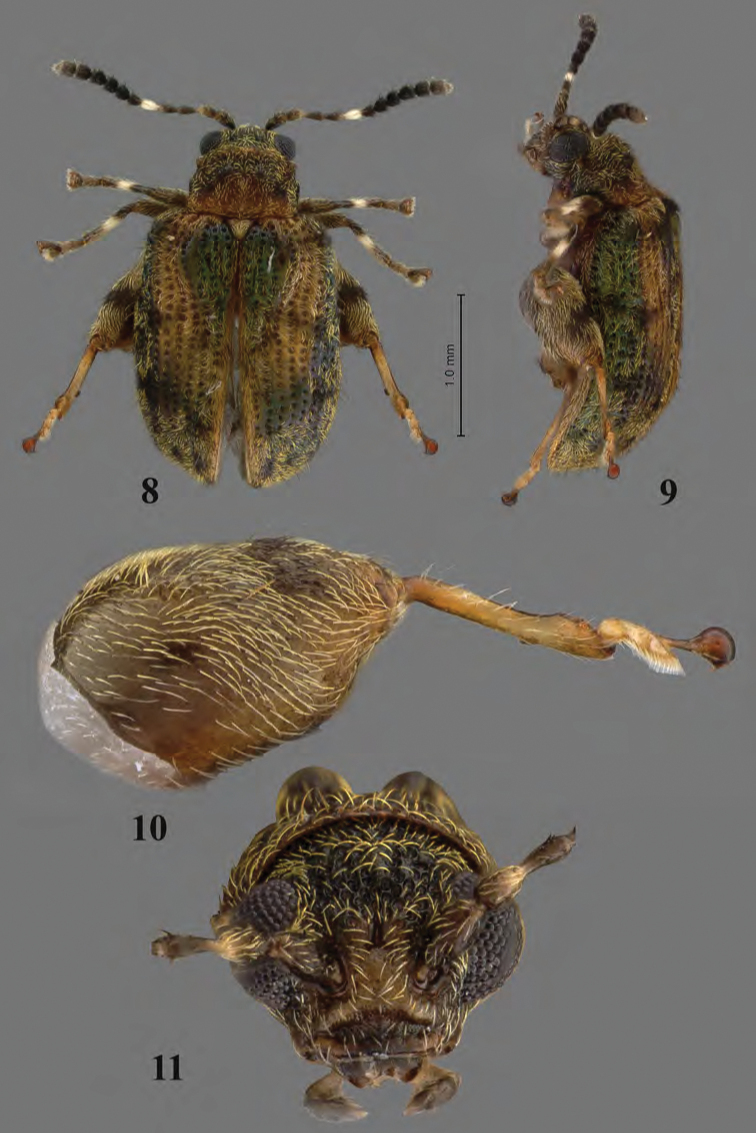
Adult *Erinaceialtica gabbysalazarae*, female **8** habitus, dorsal view **9** habitus, lateral view **10** hind leg, lateral views **11** head, frontal view.

#### Type material examined.

Holotype male: 1) Dominican Republic, Barahona Pr., El Cachote 8.XII 2014, 961m 18°03.295'N, 71°09.778'W WP-189 Leg. A. S. Konstantinov; 2) 2014.12.08 0633 (code for molecular voucher) (USNM). Paratypes with the same labels as holotype (2 USNM).

**Figures 12, 13.**
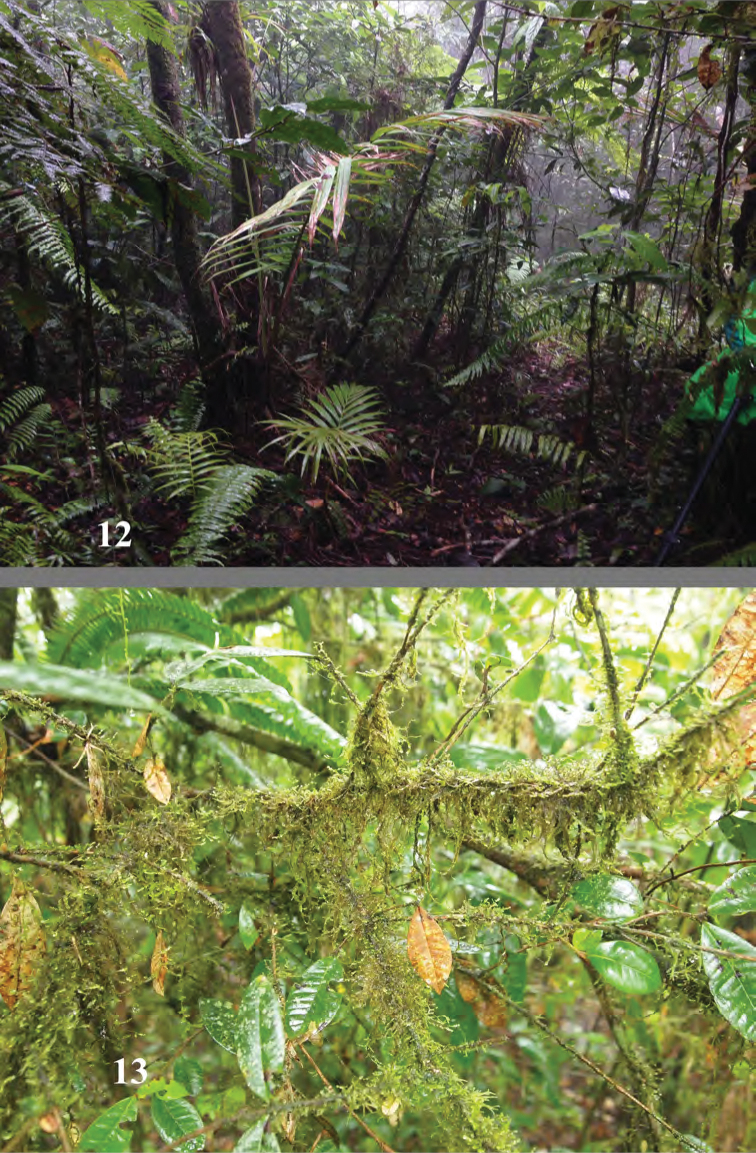
El Cachote, habitat of *Erinaceialtica gabbysalazarae*.

### Erinaceialtica janestanleyae sp. nov.

Taxon classificationAnimaliaColeopteraChrysomelidae

D4CB9F2C-9200-5670-930D-9099DBD52BC5

http://zoobank.org/FD44BB79-692A-410A-A886-CBF9A6143946

Figs 14 , 15–19 , 20–21 , Map 1

#### Description.

Body length 2.92–2.97 mm, width 1.45–1.51 mm. Vertex and part of head below vertex with antennal calli and frons same bright metallic green. Antennomere 11 slightly lighter than 9. Base and sides of pronotum same color as apex, bright metallic green. Antebasal pronotal impression absent. Elytral disc bright metallic green. Some spots on elytron appear black in part because elytral surface darker, in part because it is covered with dark setae. In dorsal view dark spots are: below basal margin lateral to scutellum, on lateral slope near middle, and another spot directly below it towards posterior. Elytra towards apex also appear darker. Ventral side of body dark brown, except last abdominal segment being lighter in color. Base of pro- and mesofemora white. Apex of pro- and mesofemora and most of pro- and mesotibiae dark brown, except small lighter ring around middle tibiae. Metafemora dark, with bronzy shine. Metatibia dark yellow. Setae on orbit and vertex yellow, directed laterally. Setae on middle of vertex short, directed towards middle, not forming a small “mohawk”. Pronotal setae directed posteriorly starting from about middle. Second row on punctures on elytral slope longitudinally impressed with setae directed laterally and ventrally from it.

**Figure 14.**
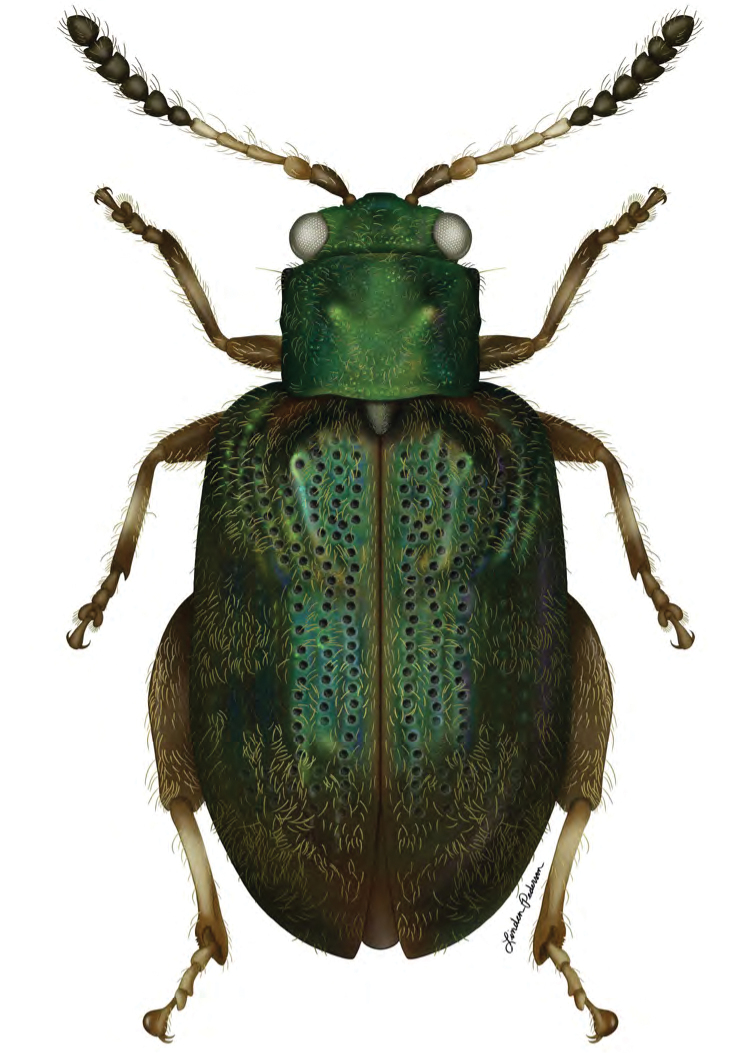
*Erinaceialtica janestanleyae* sp. nov., dorsal habitus (illustration by Linden Pederson, USNM and SEL scientific illustrator internship program, summer 2019).

#### Diagnosis.

*Erinaceialtica janestanleyae* has unique color and can be easily identified using the key at the end of the paper.

**Figures 15–19.**
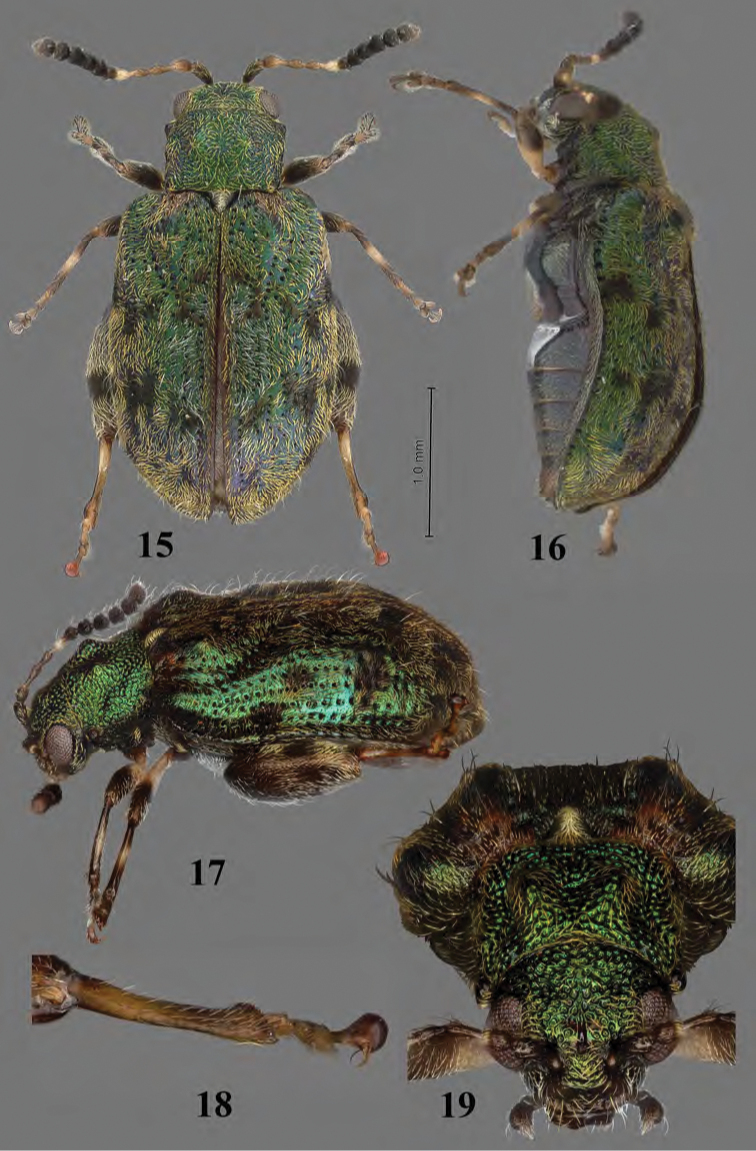
Adult *Erinaceialtica janestanleyae*, female **15** habitus, dorsal view **16** habitus, lateral view **17** habitus, three quarter view **18** hind tibia, three quarter view **19** habitus, frontal view.

#### Habitat.

**Figures 20, 21.**
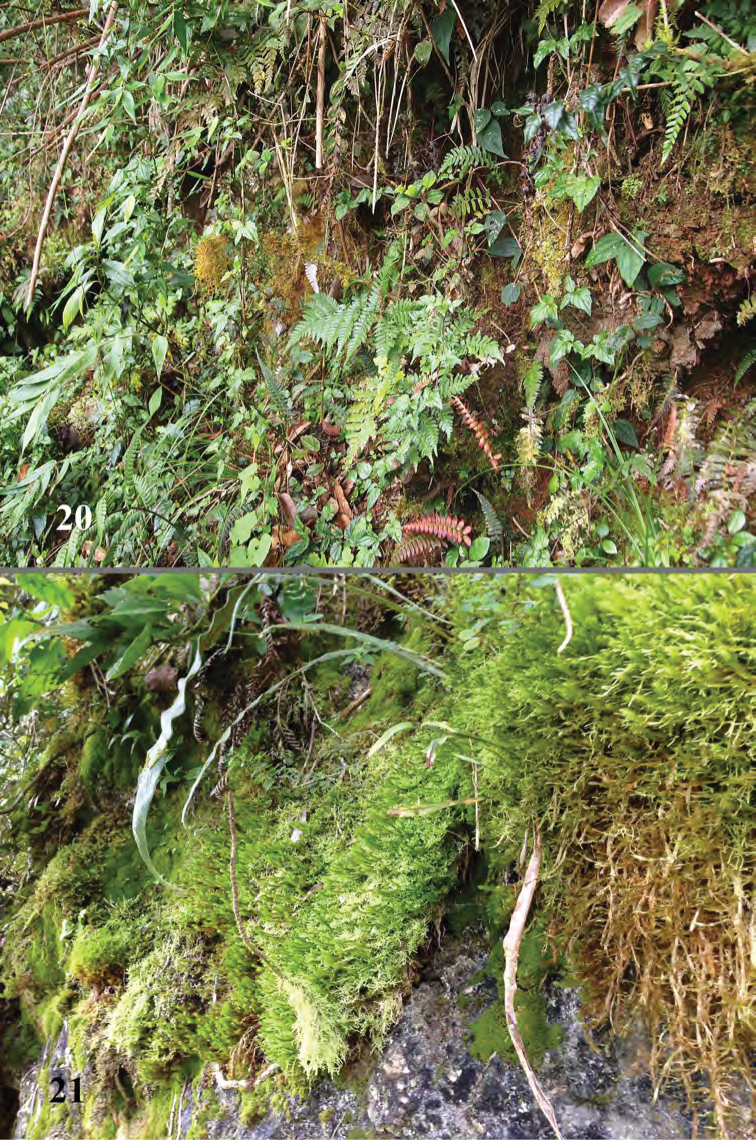
Zapoten, habitat of *Erinaceialtica janestanleyae*.

*Erinaceialtica janestanleyae* was collected in Zapoten forest (northern slopes of Sierra de Baoruco) in moss that was abundant on the sides of the road, tree trunks and branches (Figs 20, 21). Altogether about 30 gallons (= 114 L) of moss was collected in five pillowcases. Small portion of it was processed directly with Berlese extraction and the rest was sifted and then processed with Berlese. This moss collecting event revealed the largest diversity of flea beetles that included one species of *Erinaceialtica*, one species of *Kiskeya* Konstantinov & Chamorro and a species of *Andersonaltica* (Konstantinov et al. in press).

#### Etymology.

The species epithet, janestanleyae, is a matronym in honor of Jane Stanley of Bethesda, Maryland. We greatly appreciate Jane and her family’s friendship and generosity in providing access and encouragement at the Punta Cana Resort, including use of their beautiful home, Casa de los Sueños, near the Ecological Reserve.

#### Type material examined.

Holotype female: 1) Dominican Republic, Zapoten, 15.XII 2014, highest Pls, 1705 m, WP-522, 18°18.496'N, 71°41.994'W, Leg. A, Konstantinov; 2) 2014.12.15 2324 (code for molecular voucher) (USNM). Paratype female: 1) Dominican Republic, Independencia, Sierra de Baoruco, Zapoten h-1705m 15.XII.2014 WP-522, 18.19.655N, 71.41.994W, thick moss cushion on rocks trees, leg. A. Konstantinov; 2) 2014.12.15 0636 (code for molecular voucher) (USNM).

### Erinaceialtica rickstanleyi sp. nov.

Taxon classificationAnimaliaColeopteraChrysomelidae

B05F19A1-D2B7-5B81-90C1-AE056C60E084

http://zoobank.org/26EF38BD-5F0C-4054-806B-2E06BFCE4EAF

Figs 22 , 23–27 , 28–33 , 34–40 , 41–43 , 44–45 , Map 1

#### Description.

Body length 2.59–2.86 mm, width 1.45–1.78 mm. Vertex and part of head below vertex including frons same metallic blue. Antennal calli with a greenish shine. Antennomere 11 slightly lighter than 9. Base and sides of pronotum same color as apex, metallic blue and purple (some specimens have pronotum with greenish tint). Antebasal pronotal impression absent. Elytral disc metallic blue and purple. Some spots on elytron appear black in part because elytral surface darker, in part because it is covered with dark setae, these places also slightly impressed compared to rest of elytron surface. In dorsal view dark spots are: on lateral slope near middle and another spot directly below it towards posterior and one on posterior end. Elytra towards apex also appear darker. Triangular spot lateral to scutellum dark yellow. Ventral side of body dark brown, except last abdominal segment being lighter in color. Base of pro- and mesofemora light yellow. Apex of pro- and mesofemora and most of pro- and mesotibiae dark brown, except lighter poorly defined ring around middle tibiae. Metafemora dark, with bronzy shine. Metatibia dark yellow at base, brown near apex. Setae on orbit and vertex whitish, directed laterally. Setae on middle of vertex short, directed towards middle, not forming a small “mohawk”. Pronotal setae directed posteriorly starting from about middle. Second row on punctures on elytral slope longitudinally impressed with setae directed laterally and ventrally from it. Median lobe of aedeagus narrows substantially from middle to apex in ventral view, apex rounded.

**Figure 22.**
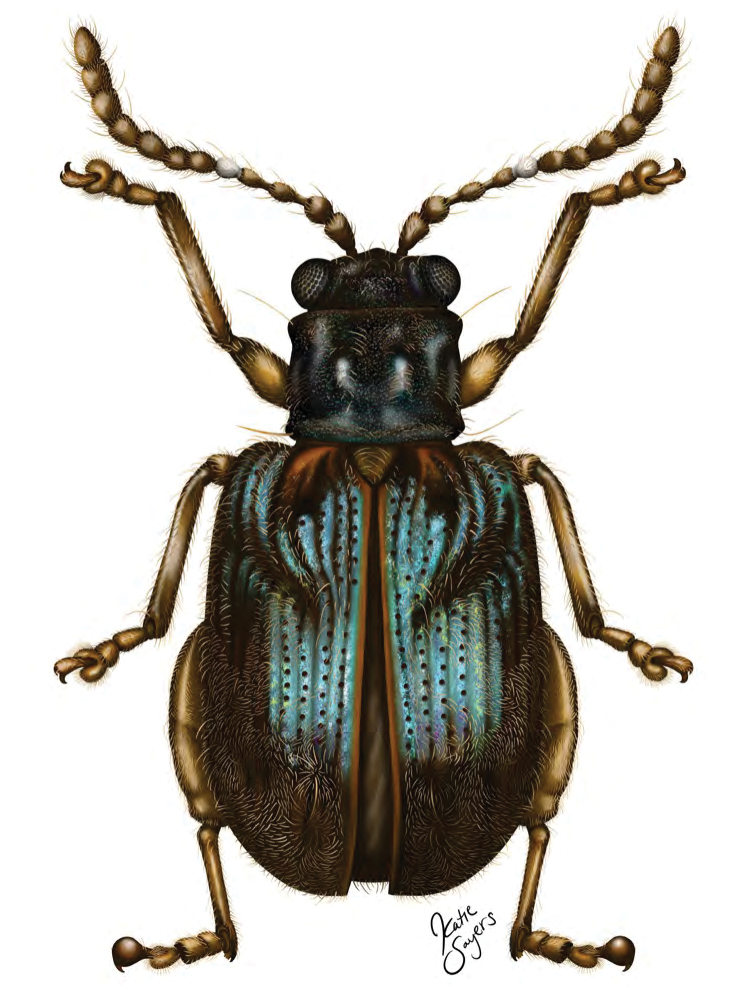
*Erinaceialtica rickstanleyi* sp. nov., dorsal habitus (illustration by Katie Sayers, USNM and SEL scientific illustrator internship program, summer 2019).

#### Diagnosis.

*Erinaceialtica rickstanleyi* has unique color and can be easily identified using the key at the end of the paper.

**Figures 23–27.**
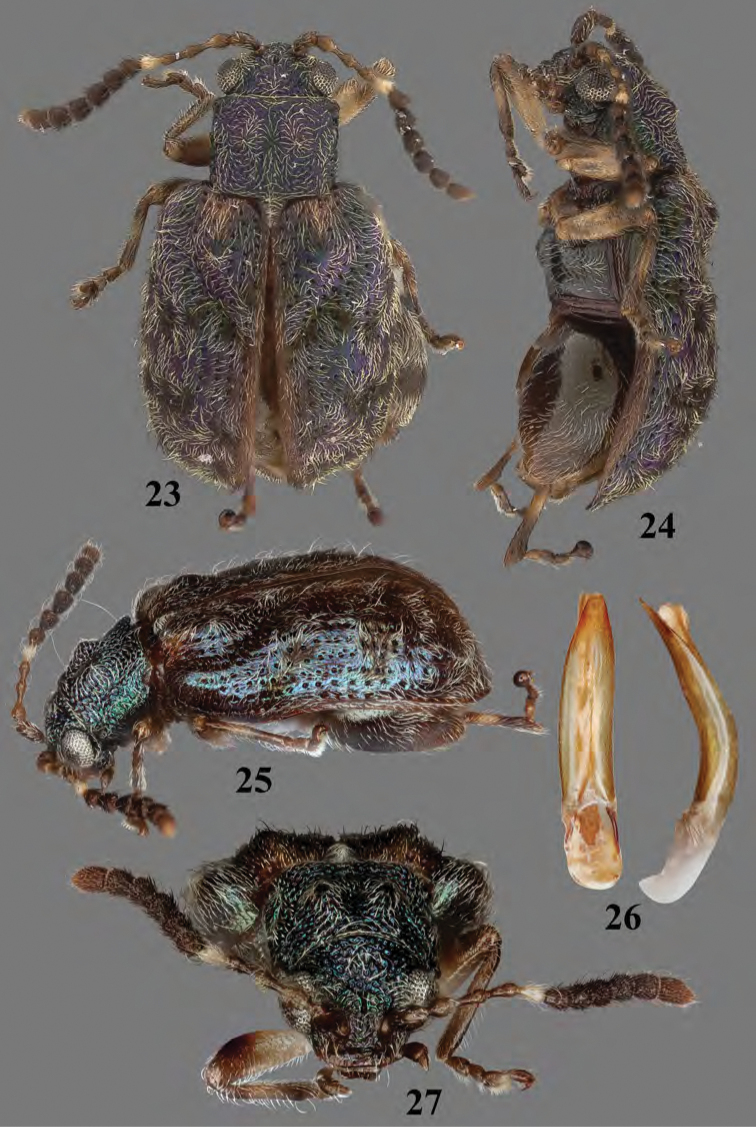
Adult *Erinaceialtica rickstanleyi***23** habitus, dorsal view **24** habitus, lateral view **25** habitus, three quarter view **26** median lobe of aedeagus, ventral and lateral views **27** habitus, frontal view.

#### Habitat.

*Erinaceialtica rickstanleyi* was collected on the southern slopes of Sierra de Baoruco in the area called Las Abejas. The site is a deep ravine situated at about 1200 m, which descends abruptly from pine forest (Figs 44, 45). Fisher-Meerow and Judd (1989) classified the area as premontane wet forest, rich in epiphytes. Moss was sampled three times in this area, in July of 2004, late June of 2005 and mid July of 2006. No moss inhabiting flea beetles were collected in 2004. In addition to *E. rickstanleyi*, *Kiskeya baorucae* Konstantinov & Chamorro-Lacayo 2006 was found in that place in 2005 and 2006.

**Figures 28–33.**
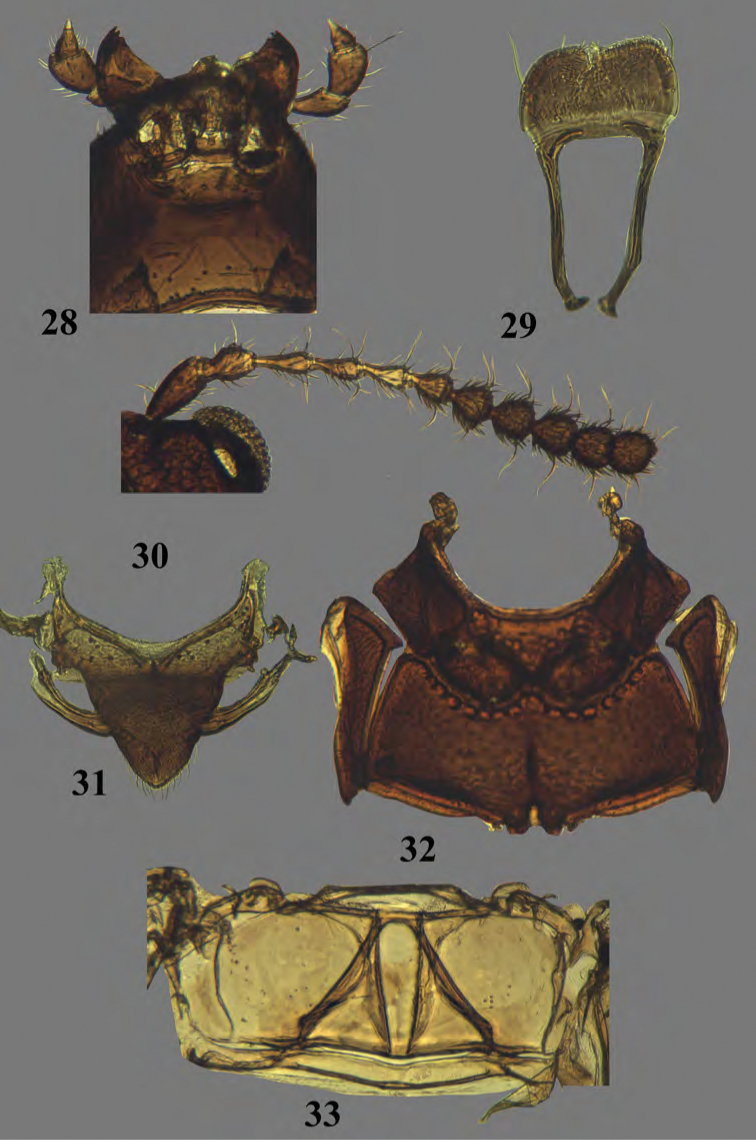
Adult *Erinaceialtica rickstanleyi*, morphological structures **28** middle part of head, ventral view **29** labrum **30** antenna **31** mesotergite **32** meso- and metasternites **33** metatergite.

#### Etymology.

The species epithet, rickstanleyi, is a patronym in honor of Rick Stanley of Bethesda, Maryland. We greatly appreciate Rick and his family friendship and generosity in providing access at the Punta Cana Resort, including use of their home, Casa de los Sueños. Rick took part in our numerous collecting trips over the years in Bolivia, Costa Rica, Dominican Republic, and Nicaragua documenting local landscapes, avian- and insect faunas with numerous photos, some of which were used in publications (e.g. Konstantinov et al. 2009).

**Figures 34–40.**
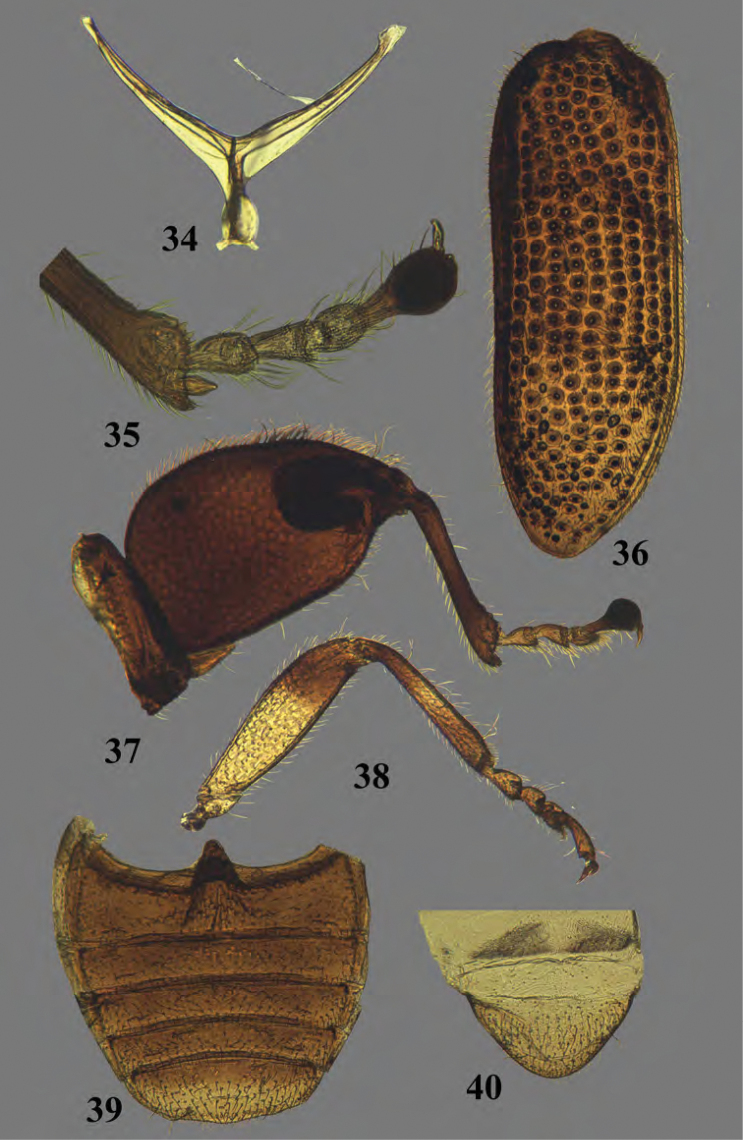
Adult *Erinaceialtica rickstanleyi*, morphological structures **34** metendosternite **35** hind tarsus **36** right elytron **37** hind leg **38** middle leg **39** abdominal ventrites, female **40** last abdominal tergite, female.

#### Type material examined.

Holotype, male: 1) Dominican Republic: Pedernales Prov., PN Sierra de Baoruco, Las Abejas 1270 m, 18°09.008'N, 71°37.338'W, 18.VI.2005, moss sifting A. Konstantinov (USNM). Paratype females 3, with the same labels as holotype (2 USNM), (1 MHND). Paratype male: Dominican Republic: Pedernales Province Sierra de Baoruco, Las Abejas forest 1230 m. 17.VII.2006 18°09.132'N, 71°37.430'W leg. A.Konstantinov (USNM).

**Figures 41–43.**
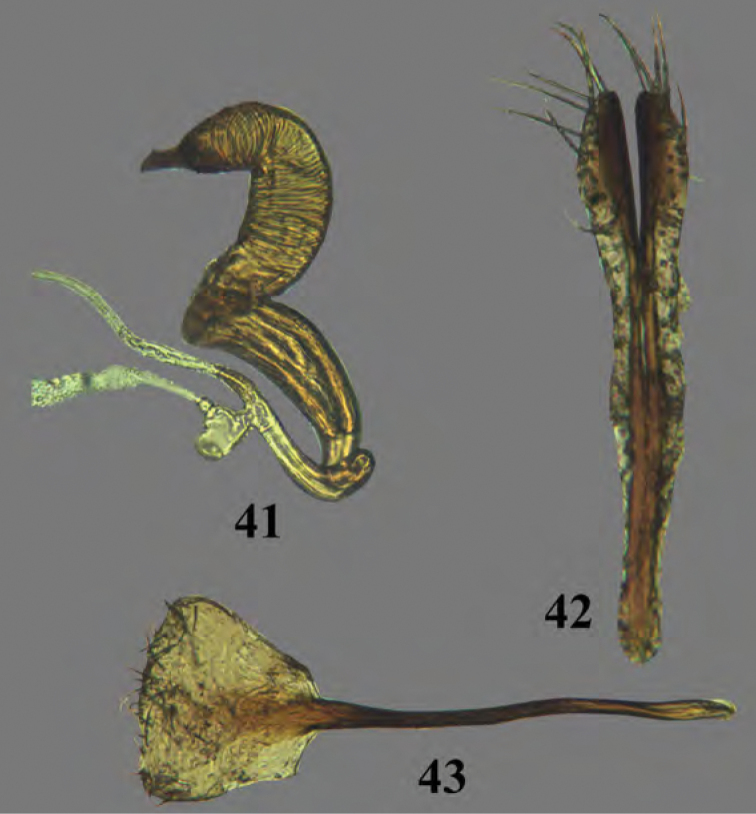
Adult *Erinaceialtica rickstanleyi*, female genitalia **41** spermatheca **42** vaginal palpi **43** tignum.

**Figures 44, 45.**
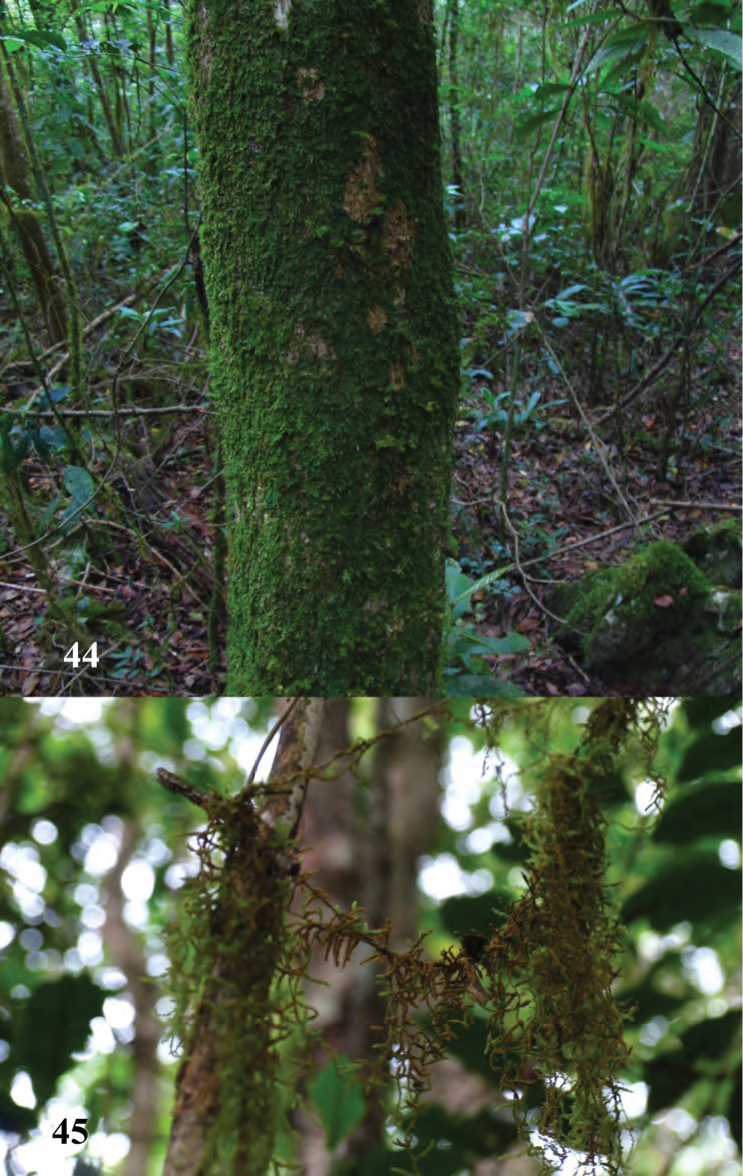
Las Abejas, habitat of *Erinaceialtica rickstanleyi*.

### Erinaceialtica rileyi sp. nov.

Taxon classificationAnimaliaColeopteraChrysomelidae

3719063D-5AE8-529D-A6EC-5768C468A65F

http://zoobank.org/0ADEC145-16A8-44DD-8AEC-67C3AA2A3930

**Figures 46, 47.**
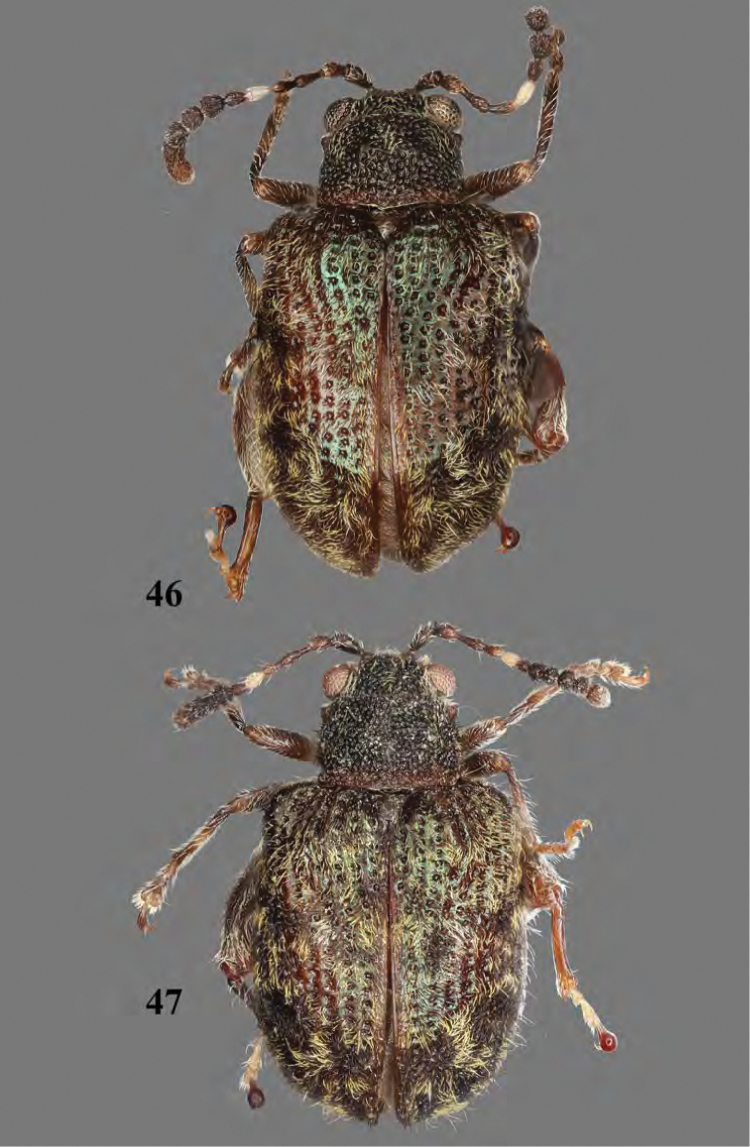
Adult *Erinaceialtica rileyi* sp. nov. **46** habitus, dorsal view, male **47** habitus, dorsal view, female.

**Figures 48–51.**
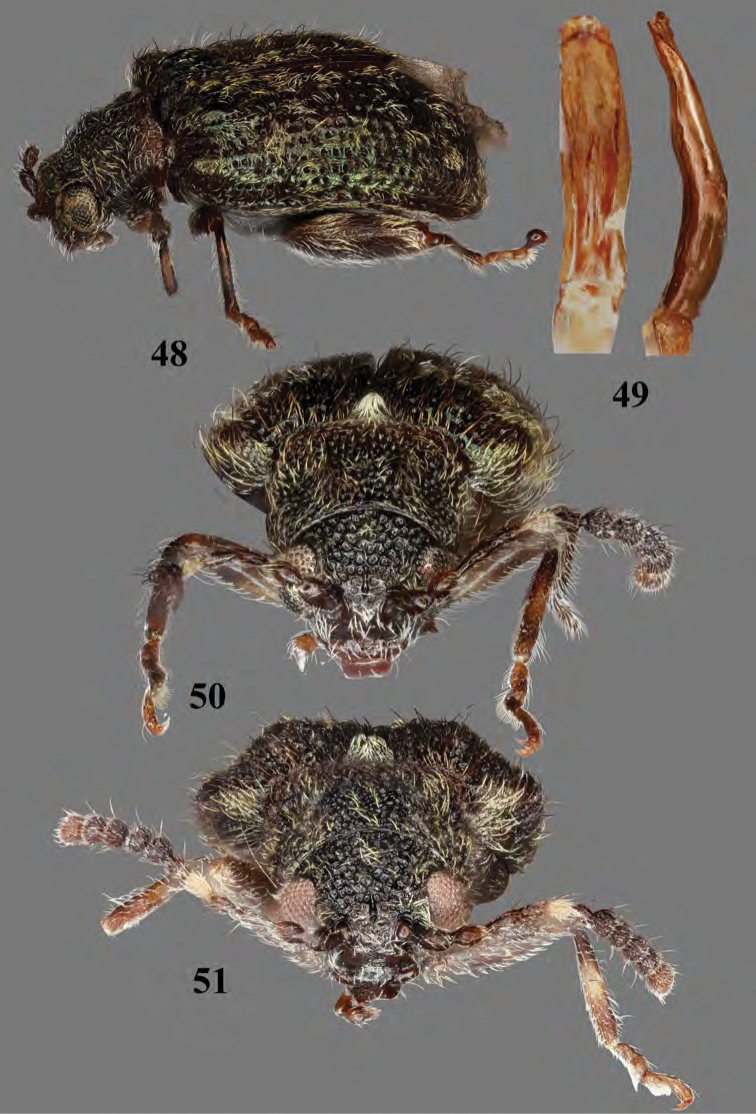
Adult *Erinaceialtica rileyi***48** habitus, three quarter view, male **49** median lobe of aedeagus, ventral and lateral views **50** habitus, frontal view, male **51** habitus, frontal view, female.

Figs 46–47 , 48–51 , Map 1

#### Description.

Body length 2.10–2.54 mm, width 1.18–1.40 mm. Vertex and part of head below vertex including frons and antennal calli black with dark greenish to blueish tint. Antennomere 11 slightly lighter than 9. Base of pronotum dark yellow, slightly lighter in color than apex. Antebasal pronotal impression absent. Elytral disc black to dark brown with dark greenish to blueish tint. Some spots on elytron appear black in part because elytral surface darker, in part because it is covered with dark setae. In dorsal view dark spots are: one spot lateral to scutellum, two spots down posteriorly near suture, and two more on posterior slope; one spot on humeral callus, two spots behind each other on lateral slope near middle, and another spot directly below it towards posterior. Elytra laterally and towards apex also appear darker. Ventral side of body dark brown, except last abdominal segment being lighter in color. Pro- and mesofemora and pro- and mesotibiae brown, with slightly lighter area on tibiae. Metafemora dark, with bronzy shine. Metatibia uniformly brown to dark amber in color. Setae on orbit and vertex whitish, denser and more vivid on orbit, directed ventrally. Setae on middle of vertex short, directed towards middle, not forming a small “mohawk”. Pronotal setae more erect, directed laterally and posteriorly. Second row on punctures on elytral slope not impressed with setae directed laterally and ventrally from it. Median lobe of aedeagus narrows gradually from middle to apex in ventral view with wide apex.

#### Diagnosis.

*Erinaceialtica rileyi* has unique color and can be easily identified using the key at the end of the paper.

#### Habitat.

Unknown.

#### Etymology.

The species epithet, rileyi, is a patronym in honor of Ed Riley, who contributed greatly to our knowledge of diversity and taxonomy of Chrysomelidae in the United States and the New World in general.

#### Type material examined.

Holotype, male: 1) Dom. Rep.: LaVega 19 km E El Rio Aug 3, 1979, C.W.O’Brien (USNM). Paratype, female, with the same labels as holotype (ERPC).

Paratype, male: Dominican Republic, La Vega, Estacion Cabanito 20 July 1996, R. Turnbow; 2) Reserva Cientifica Ebano Verde (ERPC).

### Erinaceialtica thomasi sp. nov.

Taxon classificationAnimaliaColeopteraChrysomelidae

390581D3-2418-590F-86FD-7AC380B4C87F

http://zoobank.org/D4F0C9CE-880C-4942-90D4-5F4559046D85

Figs 52–55

#### Description.

Body length 2.37 mm, width 1.24 mm. Vertex and part of head below vertex including frons and antennal calli black without apparent greenish or blueish tint. Antennomere 11 as dark as 9. Pronotum dark brown with base slightly lighter in color than apex. Antebasal pronotal impression very shallow and poorly defined. Two bumps on pronotum short with even shorter one in between them close to posterior margin. Elytral disc with two long, triangular, dark greenish to blueish metallic tint along suture. Rest of disc posteriorly dark yellow. Elytra laterally and towards apex appear darker. Ventral side of body dark brown to black, except last abdominal segment being lighter in color. Pro- and mesofemora yellow at base, dark brown for rest of its length. Pro- and mesotibiae brown, with slightly lighter area around middle. Metafemora dark, with bronzy shine. Metatibia bicolored, darker at apex, lighter at base. Setae on orbit and vertex as sparse and same color. Setae on middle of vertex short, directed towards middle, not forming a small “mohawk”. Pronotal setae directed laterally and posteriorly. Second row on punctures on elytral slope not impressed with setae directed laterally and ventrally from it. Median lobe of aedeagus narrows extensively from middle to apex, apex more or less cylindrical in ventral view. In lateral view, median lobe bends abruptly ventrally.

**Figures 52–55.**
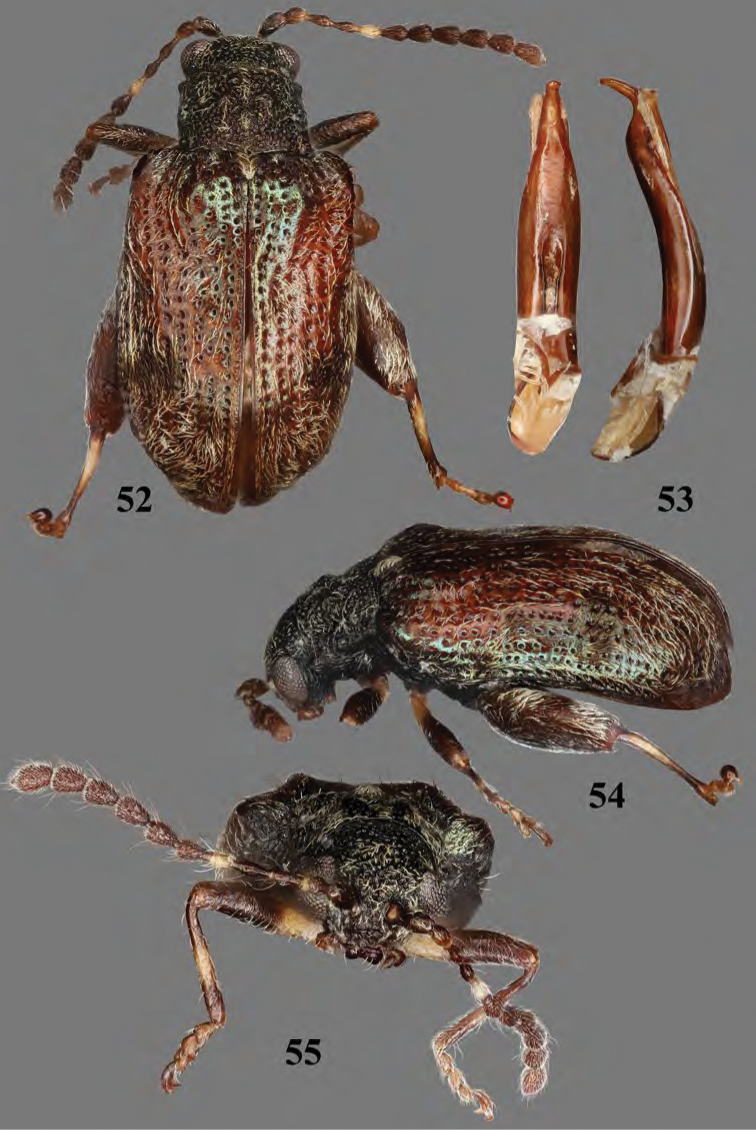
Adult *Erinaceialtica thomasi* sp. nov. **52** habitus, dorsal view **53** median lobe of aedeagus, ventral and lateral views **54** habitus, three quarter view **55** habitus, frontal view.

#### Diagnosis.

*Erinaceialtica rileyi* has unique color and can be easily identified using the key at the end of the paper.

#### Habitat.

Unknown.

#### Etymology.

The species epithet, thomasi, is a patronym in honor of late Mike Thomas, who contributed greatly to our knowledge of beetle diversity, taxonomy, and identification in the United States.

#### Type material examined.

Holotype, male; 1) Haiti Dept. Sud-Oueste, Parc National La Visite 2040–2150m, 23-V-1984 Coll. M.C. Thomas (USNM).

### Erinaceialtica albicincta

Taxon classificationAnimaliaColeopteraChrysomelidae

(Blake, 1945) comb. nov.

C0AE8BDB-7669-5252-8E78-B9988D497106

albicincta Blake 1945: 89 (Type locality. Morne La Hotte, elevation 5,000–7,800 feet, Haiti, holotype, female, MCZ); as Hadropoda. Scherer 1962: 512 as Aedmon Clark.

#### Notes.

For images see MCZ type data-base (accessed April 16, 2020): https://mczbase.mcz.harvard.edu/MediaSearch.cfm?action=search&media_id=314649,314650,314651,314652,314653

**Figures 56–61.**
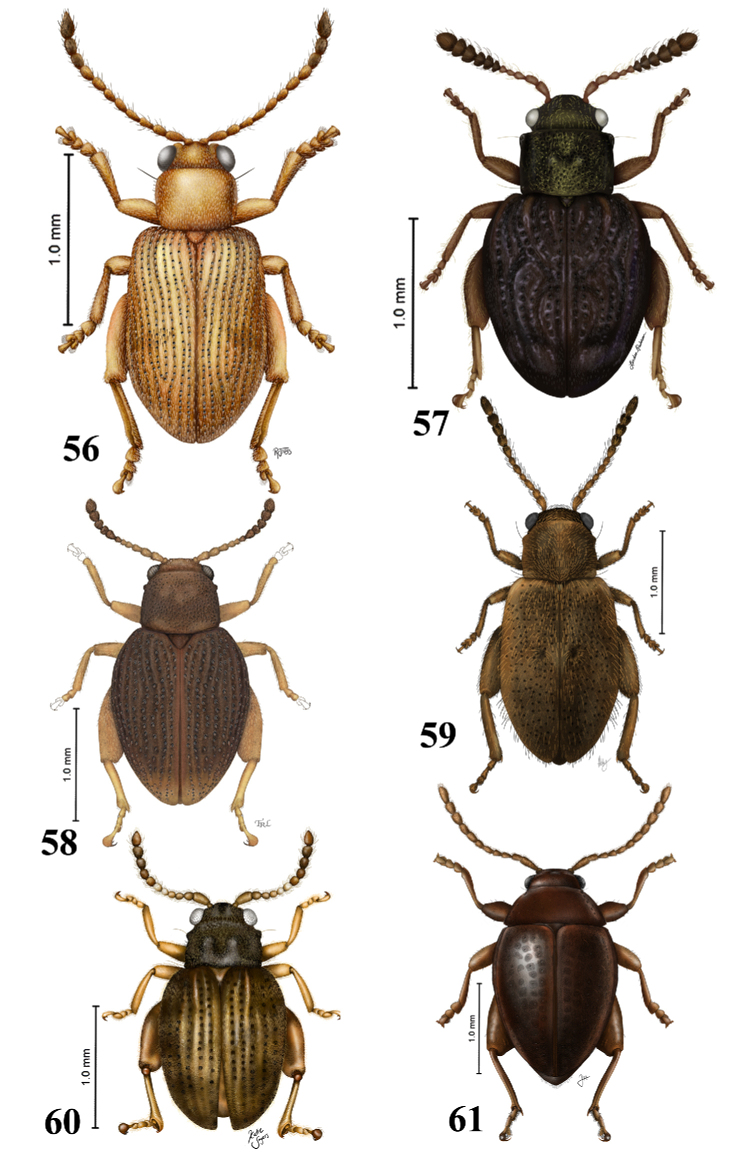
Habitus drawing of moss inhabiting Monoplatina. **56***Aedmon sericellum* Clark (illustration by Rebecca Jabs, National Museum of Natural History and Systematic Entomology Laboratory scientific illustrator internship program, summer 2016) **57***Andersonaltica villabarrancoli* Konstantinov & Linzmeier (illustration by Linden Pederson, National Museum of Natural History and Systematic Entomology Laboratory scientific illustrator internship program, summer 2019) **58***Apleuraltica curculionoides* Bechyne (illustration by Taina Litwak Systematic Entomology Laboratory) **59***Distigmoptera borealis* Blake (illustration by Abby Williams, National Museum of Natural History and Systematic Entomology Laboratory scientific illustrator internship program, summer, summer 2016) **60***Menudos maricao* Linzmeier & Konstantinov (illustration by Katie Sayers, National Museum of Natural History and Systematic Entomology Laboratory scientific illustrator internship program, summer 2019) **61***Ulrica eltoro* Konstantinov & Konstantinova (illustration by Jessica Hsiung, National Museum of Natural History and Systematic Entomology Laboratory scientific illustrator internship program, summer 2013).

### Erinaceialtica hugonis

Taxon classificationAnimaliaColeopteraChrysomelidae

(Blake, 1943a) comb. nov.

33F817F7-51EA-5256-85C0-34C7170F1A2C

hugonis Blake 1943a: 439 (type locality: Cloud forest, vicinity of Valle Nuevo, circa 6000 ft., Dominican Republic; holotype, male, MCZ); Hadropoda. Scherer 1962: 512 as Aedmon Clark.

#### Notes.

For images see MCZ type data-base (accessed April 16, 2020) https://mczbase.mcz.harvard.edu/MediaSearch.cfm?action=search&media_id=314502,314636,314637,314638,314639,314640,314641,314642,314643

### Key to *Erinaceialtica* species

**Table:** 

1	Antennomere 6 whitish	***Erinaceialtica albicincta* (Blake)**
–	Antennomere 6 dark (Fig. 2)	**2**
2(1)	Elytron bicolorous, with broad, yellow to brown oblique stripe starting from around humeral calli and extending towards suture and below (Figs 4, 46)	**3**
–	Elytron unicolorous, without broad, brownish oblique stripe starting from around humeral calli and extending towards suture and below (Fig. 15)	**5**
3(2)	Elytron with broad yellowish stripe. Dark colored part of elytron with bright metallic green lustre (Fig. 4). Median lobe of aedeagus with apical part wider than middle (Fig. 6)	***Erinaceialtica gabbysalazarae* sp. nov.**
–	Elytron with broad brownish stripe. Dark-colored part of elytron with dull metallic greenish and blueish lustre (Fig. 54). Median lobe of aedeagus with apical part as wide or narrower than middle (Figs 46, 53)	**4**
4(3)	Median lobe of aedeagus narrows extensively from middle to apex, apex more or less cylindrical in ventral view. In lateral view, median lobe bends abruptly ventrally (Fig. 53)	***Erinaceialtica thomasi* sp. nov.**
–	Median lobe of aedeagus more or less parallel sided from middle to apex, apex flat in ventral view. In lateral view, median lobe bends slightly dorsally	***Erinaceialtica hugonis* (Blake)**
5(2)	Head, pronotum, and elytron bright metallic green (Fig. 17).	***Erinaceialtica janestanleyae* sp. nov.**
–	Head, pronotum, and elytron mostly black or dark metallic blue or green with a few light-green spots (Fig. 25)	**6**
6(5)	Base of elytron with dark-yellow spot lateral to scutellum (Fig. 23). Median lobe of aedeagus narrows substantially from middle to apex in ventral view (Fig. 26)	***Erinaceialtica rickstanleyi* sp. nov.**
–	Base of elytron without dark-yellow spot lateral to scutellum (Figs 46, 47). Median lobe of aedeagus narrows gradually from middle to apex in ventral view (Fig. 49)	***Erinaceialtica rileyi* sp. nov.**


## Supplementary Material

XML Treatment for Erinaceialtica

XML Treatment for Erinaceialtica gabbysalazarae

XML Treatment for Erinaceialtica janestanleyae

XML Treatment for Erinaceialtica rickstanleyi

XML Treatment for Erinaceialtica rileyi

XML Treatment for Erinaceialtica thomasi

XML Treatment for Erinaceialtica albicincta

XML Treatment for Erinaceialtica hugonis
